# Anionic polymers amplify electrokinetic perfusion through extracellular matrices

**DOI:** 10.3389/fbioe.2022.983317

**Published:** 2022-09-26

**Authors:** Joseph C. Walker, Ashley M. Jorgensen, Anyesha Sarkar, Stephen P. Gent, Mark A. Messerli

**Affiliations:** ^1^ Department of Biology and Microbiology, South Dakota State University, Brookings, SD, United States; ^2^ Department of Mechanical Engineering, South Dakota State University, Brookings, SD, United States

**Keywords:** electrokinetic perfusion, extracellular matrix, anionic polymers, electro-osmosis, interstitial flow

## Abstract

Electrical stimulation (ES) promotes healing of chronic epidermal wounds and delays degeneration of articular cartilage. Despite electrotherapeutic treatment of these non-excitable tissues, the mechanisms by which ES promotes repair are unknown. We hypothesize that a beneficial role of ES is dependent on electrokinetic perfusion in the extracellular space and that it mimics the effects of interstitial flow. *In vivo*, the extracellular space contains mixtures of extracellular proteins and negatively charged glycosaminoglycans and proteoglycans surrounding cells. While these anionic macromolecules promote water retention and increase mechanical support under compression, in the presence of ES they should also enhance electro-osmotic flow (EOF) to a greater extent than proteins alone. To test this hypothesis, we compare EOF rates between artificial matrices of gelatin (denatured collagen) with matrices of gelatin mixed with anionic polymers to mimic endogenous charged macromolecules. We report that addition of anionic polymers amplifies EOF and that a matrix comprised of 0.5% polyacrylate and 1.5% gelatin generates EOF with similar rates to those reported in cartilage. The enhanced EOF reduces mortality of cells at lower applied voltage compared to gelatin matrices alone. We also use modeling to describe the range of thermal changes that occur during these electrokinetic experiments and during electrokinetic perfusion of soft tissues. We conclude that the negative charge density of native extracellular matrices promotes electrokinetic perfusion during electrical therapies in soft tissues and may promote survival of artificial tissues and organs prior to vascularization and during transplantation.

## Introduction

During electrical therapies of nonexcitable tissues, the underlying cells and supporting extracellular matrix are exposed to applied electric fields (EFs). These therapies have been applied to heal chronic epidermal wounds, delay degeneration of articular cartilage and promote transplantation of tissues (Politis et al., 1989; Mont et al., 2006; Frykberg and Banks, 2015; Houghton, 2017; Gomes et al., 2018). Healing of epidermal wounds is thought to be associated with EF-directed migration of surrounding keratinocytes into the wound bed (Nishimura et al., 1996; Fang et al., 1998; Zhao, 2009). In this regard, EFs expose cell surface macromolecules to electrokinetic forces, primarily electro-osmotic flow (EOF) and electrophoresis (McLaughlin and Poo, 1981; Allen et al., 2013; Sarkar et al., 2019). The net electrokinetic force leads to accumulation of cell surface macromolecules to one of the electrical poles and is hypothesized to modify cell polarity and influence the direction of cellular migration or growth. EOF toward the cathode generates shearing flow (Huang et al., 2009; Graham et al., 2013) and can be reduced near the cell surface with neutral viscous polymers, leading to reversal of the direction of cell migration (Kobylkevich et al., 2018). While a majority of research effort has emphasized understanding the role of DC EFs in polarizing cells, the effects of the EFs on the surrounding extracellular matrix have been largely ignored (McCaig et al., 2005; Zhao, 2009; Messerli and Graham, 2011; Funk, 2015).

EFs also induce electrokinetic forces in the extracellular matrices surrounding cells (Garcia et al., 1996; Sarkar and Messerli, 2021) and may help overcome limitations to mass transfer during treatment of degenerative diseases, tissue engineering, and tissue transplantation. In the absence of sufficient blood flow, mass transfer is limited to diffusion, reducing the supply of nutrients and accumulating metabolic wastes that lead to early cell mortality. Successful transplantations of avascular tissues are limited to structures that are small, thin, or have low cellular density, e.g. smaller diameter pancreatic islets (Lehmann et al., 2007; Suszynski et al., 2014), corneas (Tan et al., 2012), cardiac patches (Taylor et al., 2014), epidermis (Böttcher-Haberzeth et al., 2010), or cartilage (Gomoll and Minas, 2014; Bugbee et al., 2016). *In vivo*, some tissues overcome diffusion limitations by using interstitial flow, extracellular fluid flow between cells and within the extracellular matrix. For example, during normal use of cartilage, cycles of compression and decompression promote interstitial flow, while other tissues generate interstitial flow through hydrostatic and osmotic pressure differences between the vasculature, extracellular space, and lymphatic vessels (Swartz and Fleury, 2007). We hypothesize that EFs applied to nonexcitable tissues promote tissue survival and health by promoting EOF through the extracellular matrix and mimicking the effects of interstitial flow.

Extracellular matrices contain a mixture of proteins, proteoglycans, and glycosaminoglycans. The glycans possess a high degree of negative charge enabling them to attract water and provide mechanical support. Articular cartilage is 15%–22% collagen and 10–15% glycans (Sophia Fox et al., 2009) while fibrocartilage (knee meniscus) is 21% collagen and 5% glycans (Makris et al., 2011). Glycans are found in lower amounts in skin and tendons (Kannus, 2000; Lee et al., 2016), however, they are concentrated in the narrow intercellular spaces of the two deepest cellular layers of the epidermis (Tammi et al., 1988). The net negative charge of these glycans should promote EOF in the presence of applied EFs. We test this hypothesis by comparing EOF rates through matrices of gelatin alone, and through matrices of gelatin mixed with anionic polymers. We also compare reduction of cell mortality at different applied voltages and model thermal changes to explore the scope that EFs may promote mass transfer *in vivo* and during tissue engineering. Our results support the hypothesis that native anionic macromolecules amplify EOF and reduce cellular mortality during EF application.

## Materials and methods

### Matrix construction

Gelatin was prepared at the listed concentrations by dissolving 300 bloom Type A, porcine gelatin (Sigma-Aldrich, St. Louis MO) in sterile, modified Ringer’s (in mM 145 NaCl, 3 KCl, 2 CaCl_2_, 25 HEPES pH 7.4) with glucose (5 mM). Gelatin was covalently cross-linked by mixing with 0.01% microbial transglutaminase, final concentration (Moo Gloo TI Transglutaminase, Modernist Pantry, Eliot, ME). Immediately after mixing, the gelatin-transglutaminase was loaded into IBIDI channel slides (IBIDI μ-slide VI uncoated, Fitchburg, WI) to allow crosslinking, first at ambient temperature for 0.5 h, and then at 37°C for an additional 1.5 h.

Matrices comprised of mixtures of gelatin and anionic polymers were made by combining concentrated stock solutions of the matrix materials dissolved in modified Ringer’s. Stock solutions of polyacrylic acid and polystyrene sulfonic acid (10^3^ kD, Polysciences, Warrington, PA), sodium dextran sulfate (500 kD, Alfa Aesar, Tewksbury, MA), hyaluronic acid (10^3^ kD R&D Systems, Minneapolis, MN) sodium carboxymethyl cellulose (250 kD), heparin (Thermofisher, Waltham MA), and pectin (Sigma-Aldrich, St. Louis, MO) were pH neutralized before mixing with gelatin. Transglutaminase was added to the mixtures to crosslink the gelatin as described above.

### Electro-osmotic mobility determination

Electro-osmotic flow was determined by tracking the movement of neutral, fluorescent dextrans through IBIDI-filled channels. A mini-electrophoretic chamber consisted of using a DC power supply (Lambda Electronics Inc., Melville, N.Y.) applying 2.3 mA through the artificial matrix-filled IBIDI channels. Time lapse images were collected to track movement of the dye front of 100 µM Texas Red dextrans (3, 10 or 70 kDa) with an Olympus FV1200 Confocal system mounted on an Olympus IX81 inverted microscope. Timelapse imaging of the moving dye front was performed until the dye front reached the center of the field of view. The electric field was then switched off to acquire an equal number of images to track diffusion of the dye front in order to remove diffusion from the EOF measurement. Three replicates of the dye front movement were imaged for each channel and a minimum of three channels were studied for each mixture. Tracking of the dye front was performed with a Matlab script (MathWorks, Natick MA) based on determining average movement of a selected range of pixel intensities at the dye front. Image stacks were converted to BMP formatted images prior to analysis in Matlab.

DC electric fields were applied using a programmable Lambda LLS 6120 power supply and Ag/AgCl electrodes, connected to the matrix-filled channels using 2% agarose bridges filled with modified Ringer’s. This configuration enabled electrical connection to the experimental matrices without exposing them to electrode products.

The electrical resistance of the channels varied with the composition and density of the matrix. Conductivity of modified Ringer’s and culture media was determined using a YSI 3200 conductivity instrument (Yellow Springs, OH). Electrical resistance of the different matrices in the IBIDI channels was assessed using a Metex multimeter (M-4640A Toronto, Canada) with Ag/AgCl electrodes immersed in the IBIDI wells. The applied electric field was determined for each matrix based on its resistance and applied current density, and used to convert electro-osmotic flow (units of nm/s) to electro-osmotic mobility (units of nm/s/V/m).

### Assay for cell mortality

CHO cells were maintained in long term culture of Dulbecco’s Modified Eagle’s Medium (DMEM, Gibco, Waltham, MA), with 10% FBS and 1% Penicillin-Streptomycin. Before 3D culture, cells were trypsinzed, rinsed of trypsin, and suspended in 2 ml of sterile, modified Ringer’s before seeding into artificial matrices. Cells were allowed to settle for 0.5 h at 37°C, allowing gravity to concentrate them at the bottom of the sterile Eppendorf tube. At 37°C, concentrated cells (0.5 ml) were mixed with 0.4 ml of concentrated matrix and 0.1 ml transglutaminase to make a final concentration of 0.01% enzyme. After brief and gentle mixing, a small volume of the mixture was immediately loaded into the 30 µL volume of the IBIDI channels and allowed to gel for 0.5 h at ambient temperature before warming to 37°C for an additional 1.5 h. Final matrices used for viability assays consisted of 2% gelatin alone, or 1.5% gelatin mixed with 0.5% polyacrylate. EOF was used to rinse transglutaminase from the crosslinked matrices and DC EFs were applied to IBIDI channels as described (Sarkar and Messerli, 2021). EOF was applied in the presence of modified Ringer’s-filled wells for 40 min. Rinsing of the crosslinked matrices was repeated a second time with phenol red free DMEM to further remove the enzyme and also perfuse the matrices with culture media. At this time point (day 0), cells in three channels were exposed to EOF containing propidium iodide (PI) to stain and assess the relative number of necrotic cells at the beginning of the experiment. PI labelled cells were imaged with an Olympus confocal microscope to assess mortality.

Other channels were assembled from the same batch of cells and matrix to monitor cell mortality after 48 h in the absence and presence of EOF. EFs were applied to cells using 9 V battery banks, in series with resistance substitution boxes (RS-500, Elenco, Wheeling, IL), and Ag/AgCl electrodes immersed in Ringer’s-filled beakers and connected to IBIDI channels with agarose bridges to reduce contamination by electrode products (Sarkar and Messerli, 2021). IBIDI channel slides were mounted on an aluminum block set to 37°C to reduce thermal changes that may arise due to ohmic heating. Current was monitored and adjusted regularly to maintain the EF strength. Phenol red free DMEM was refreshed every 12 h in experimental chambers during the low rate electrokinetic perfusion experiments and was refreshed every 6–8 h for the high rate experiments. After 48 h, Luer lock components were removed and matrices were stained with propidium iodide using EOF as described above. Two fluorescent images were taken at the inlet, center, and outlet of each cell-filled channel (totaling six images) using confocal microscopy. Images were acquired 30 µm above the bottom of the IBIDI channel to ensure a uniform brightness from the stained cells. The images were analyzed using ImageJ (Rasband, 1997-2018) to assess percent area of propidium iodide fluorescence. Statistical significance was determined using a one-tailed *t*-test unless otherwise noted.

### Thermal modeling

The thermal effects of electrical stimulation were simulated using a three-dimensional, implicit, segregated fluid temperature computational fluid dynamics (CFD) model. The follow equation is the governing equation for thermal energy transport:∇(k∇T)+*σ*( $\vec{E}\vec{E}$ ) = 0

The model consisted of a stationary fluid neglecting the slower effects of convection and viscous dissipation. Thermal contributions due to current density and channel dimensions were emphasized. The first term describes the thermal energy transfer due to thermal diffusion where k is thermal conductivity and T is temperature. The second term shows the joule heating source term that is due to the applied electric field where σ is electrical conductivity and $\vec{E}$ is the electric field vector.

Electrodynamic settings allowed for modeling of the electric currents within the electrically conductive domains. The current density was clamped for exploring different electric field strengths to determine the ohmic heating through the experimental IBIDI chamber and through an arrangement of cylinders intended to represent human tissues. Heat dissipation via thermal conduction was allowed to occur with the immediate surroundings. The narrow IBIDI chamber consisted of a channel with dimensions of 1.7 cm by 0.38 cm by 0.04 cm with its base adjacent to an aluminum block at 37°C similar to the experimental conditions described above. The thermal conductivity used for aluminum is 237 W/(m•K). A second geometry was used to mimic the application of an electric field through a larger soft tissue, and consisted of a cylinder of avascular tissue with thermal conductivity of 0.21 W/(m•K) reported for cartilage (Moghadam et al., 2015) that was 5 cm diameter and 5 cm long. Smaller cylindrical channels, 1 mm, 3 mm, and 10 mm, were modeled through the center of the larger cylinder to constrain current flow by limiting the electrical conductivity of their outer walls to 0 S/m. In the center, electrical conductivity was set at 1.38 S/m. Computer-aided design (CAD) representations of several idealized geometries were created in SolidWorks 29 (Dessault Systems, Waltham, MA). These CAD representations were imported into CFD simulations, which were developed and analyzed using Star-CCM+ 15.02.007 CFD software (Siemens, Munich, Germany). In this software, the geometry was discretized into an array of volumetric cells to create a mesh for determining the numerical solution.

## Results

Anionic macromolecules are a significant component of extracellular matrices (ECMs) in cartilage, dermis, and other connective tissues. Greater negative charge densities are found on the glycosaminoglycans (GAGs), e.g. chondroitin sulfate, keratan sulfate and hyaluronate, than the naked proteins of ECMs, Table 1. However, some GAGs are covalently attached to proteins to make highly charged proteoglycans like aggrecan and versican, increasing their average charge to mass ratio by 3-4-fold. (Wight, 2002; Chandran and Horkay, 2012). Construction of artificial matrices with native charge was first attempted by testing the gelation of mixtures of gelatin with hyaluronate and other anionic polymers, Table 1, to lower experimental costs. Gelatin successfully gelled with polyacrylate, carboxymethyl cellulose, hyaluronate, and heparin. However, 1.5% gelatin did not form stable matrices with 0.5% polystyrene sulfonate, pectin, or dextran sulfate.

**TABLE 1:** Comparison of charge to mass ratio for selected macromolecules and polymers.

	Substance	M_r_ (Da)	Relative charge	Charge to Mass ratio (kDa^−1^)
PROTEIN^a^	Aggrecan	2.6×10^5^	−290	−1.1
	Versican	3.7×10^5^	−316	−0.8
	Fibronectin	2.7×10^5^	−65	−0.2
	Collagen (αI+αII)	9.4–9.5×10^4^	8–18	0.1–0.2
GAG^b^	Chondroitin sulfate	463	−2	−4.3
	Keratan sulfate II	481–561	−1 or −2	−1.8–−4.1
	Hyaluronate (HA)	379	−1	−2.6
OTHER ANIONIC POLYMERS^b^	Polyacrylate (PA)	72	−1	−13.9
	Dextran sulfate	259	−2.3	−8.9
	Heparin	572	−4	−7.0
	Pectin	194	−1	−5.2
	Polystyrene sulfonate	200	−1	−5.0
	Carboxymethyl cellulose	240	−1.2	−5.0

a Relative charge of naked proteins was determined at pH 7.4.

b The M_r_ for GAGs, and other anionic polymers are listed according to their smallest repeating subunit. Aggrecan (P16112), Versican (P13611), Fibronectin (P02751), Collagen I (P02452, P08123). Protein name (Uniprot accession ID).

### Electrokinetic perfusion through ECMs

**FIGURE 1:**
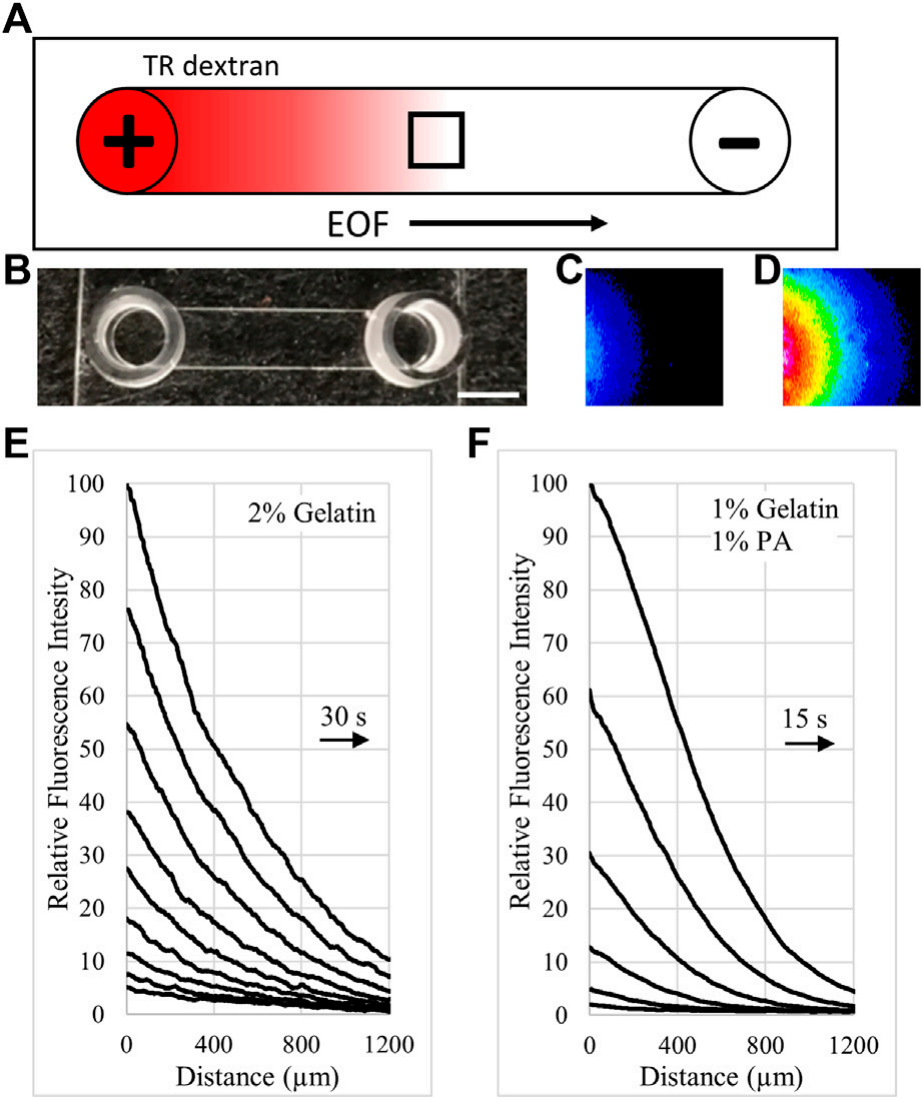
Tracking EOF rates with neutral fluorescent dextran. **(A)**. Illustration (top view) of an IBIDI channel with fluorescent, neutral Texas Red (TR) dextran loaded into the anode (+) well. The dye front moved with EOF toward the cathode (-) and was tracked to calculate EOF rate. The small black box indicates the field of view during imaging. **(B)**. Photo of an empty IBIDI channel with scale bar = 4 mm. **(C)**. Pseudocolor image of the dye front entering the left side of the field of view for a 1% gelatin, 1% PA gel. The pseudocolor scale uses the colors of the rainbow with dim fluorescence (blue) and bright fluorescence (red) **(D)**. Pseudocolor image of the dye front acquired 45 s after the image in **(C)**. **(E)**. Relative intensity profiles of the cathode moving dye front through 2% crosslinked gelatin are reported in 30 s intervals. Right pointing arrow indicates the direction of the moving dye front. **(F)**. Relative intensity profiles of the dye front through a 1% gelatin, 1% polyacrylate mixture are covering greater distance in 15 s intervals. Arrow indicates the direction of the moving dye front.

Electrokinetic perfusion was induced by applying EFs to artificial matrices comprised of different concentrations of gelatin alone or gelatin mixed with anionic polymers. IBIDI channel slides were arranged as mini-electrophoretic chambers and EOF was measured while current was clamped to provide an EF near 1 V/mm. Neutral Texas Red dextran was added to the anode well and migrated toward the cathode in the presence of an applied EF due to EOF, Figure 1A. An empty IBIDI channel, Figure 1B is shown next to two images of the dye front acquired in a mixture of 1% crosslinked gelatin with 1% PA, Figures 1C, D. The image in Figure 1D is acquired 45s after the image in Figure 1C as the dye front migrates to the right. The fluorescence intensity profiles were assessed at the dye front as the average intensity across the entire image to determine EOF rate. In 2% crosslinked gelatin, EOF progressed significantly slower, Figure 1E, than EOF in a mixture of 1% crosslinked gelatin with 1% polyacrylate (PA), Figure 1F.

**FIGURE 2:**
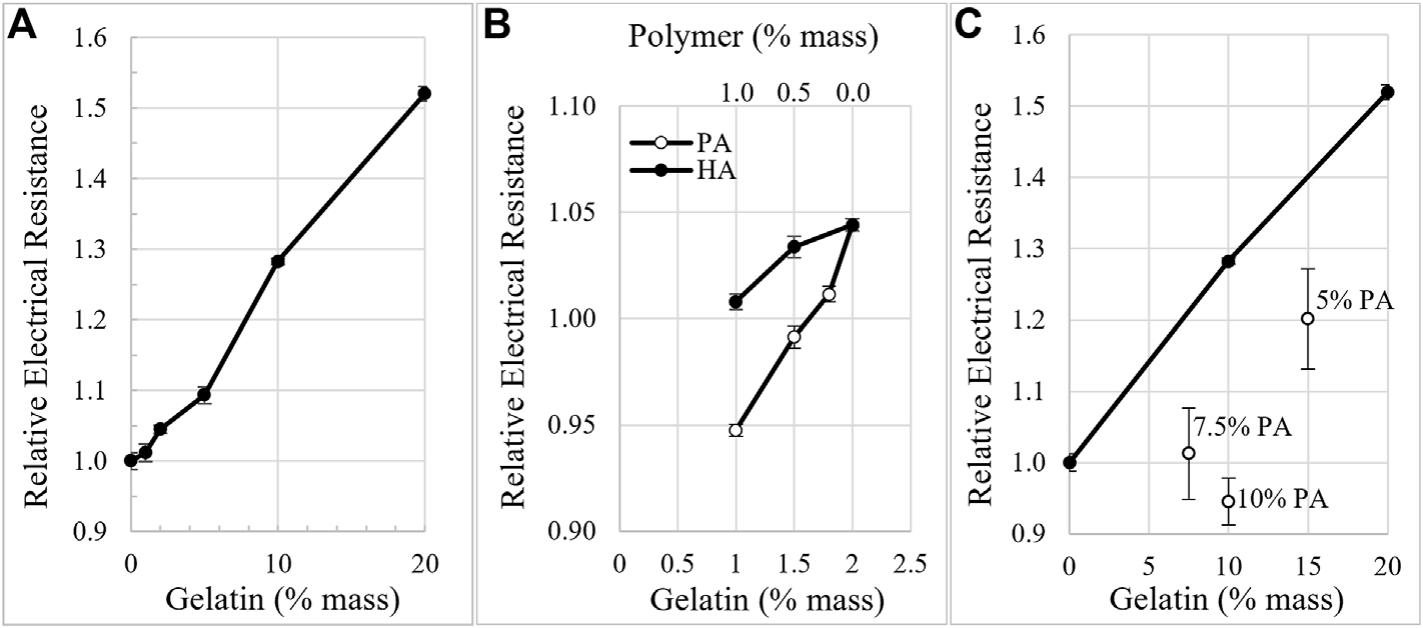
Matrix density and composition influence electrical resistance. **(A).** Electrical resistance of gelatin-filled IBIDI channels in Ringer’s, increases with gelatin concentration. **(B)**. Electrical resistance of the matrices in Ringer’s decreases with increasing amounts of anionic polymers compared to a similar percent weight of gelatin alone. 2% was the total combined weight percent of the mixtures of HA and PA with gelatin. **(C)**. Electrical resistance of high concentrations of gelatin alone (•) decreases when higher concentrations of PA are added.

Composition of the extracellular matrices in Ringer’s solution influenced electrical resistance of the matrix-filled IBIDI channels. On average, the relatively uncharged gelatin significantly increased electrical resistance of the channels by 2.4% for each 1% increase in gelatin concentration, Figure 2A. Addition of high molecular weight hyaluronate (HA) to gelatin to make 2% by weight mixtures of 1.5%:0.5% and 1%:1%, gelatin:HA, possessed lower electrical resistance compared to 2% gelatin alone, Figure 2B. Mixtures of gelatin with PA to make 2% by weight mixtures possessed even lower resistances at the same ratios compared to the gelatin:HA mixtures Figure 2B. Electrical resistance of the matrices was also decreased at higher concentrations. Mixture ratios of 7.5%:7.5% and 10%:10%, gelatin:PA, possessed electrical resistances similar to Ringer’s solution alone and 15%:5% gelatin:PA possessed electrical resistance less than half as large as 20% gelatin alone, Figure 2C.

Electrical resistance measurements were also performed on matrices undergoing electrokinetic perfusion for 48 h to ensure that resistance did not change over time and that PA was not removed from the matrices during the cellular experiments. Electrical resistance of 2% gelatin did not change significantly over the 2 days (*p* > 0.5 for both comparisons, day 0 to day 1 and day 0 to day 2). Electrical resistance of 1.5%:0.5%, gelatin:PA, did not change significantly between day 0 and day 1 (*p* > 0.2), but did decrease by 2.5% on average by day 2 compared to day 0 (*p* < 0.01). This may indicate that the PA remained in the matrix but that less conductive regions of the matrix were being removed by electrokinetic forces. PA may have interfered with transglutaminase during crosslinking of gelatin leading to a small loss of gelatin molecules during the experiment.

**FIGURE 3:**
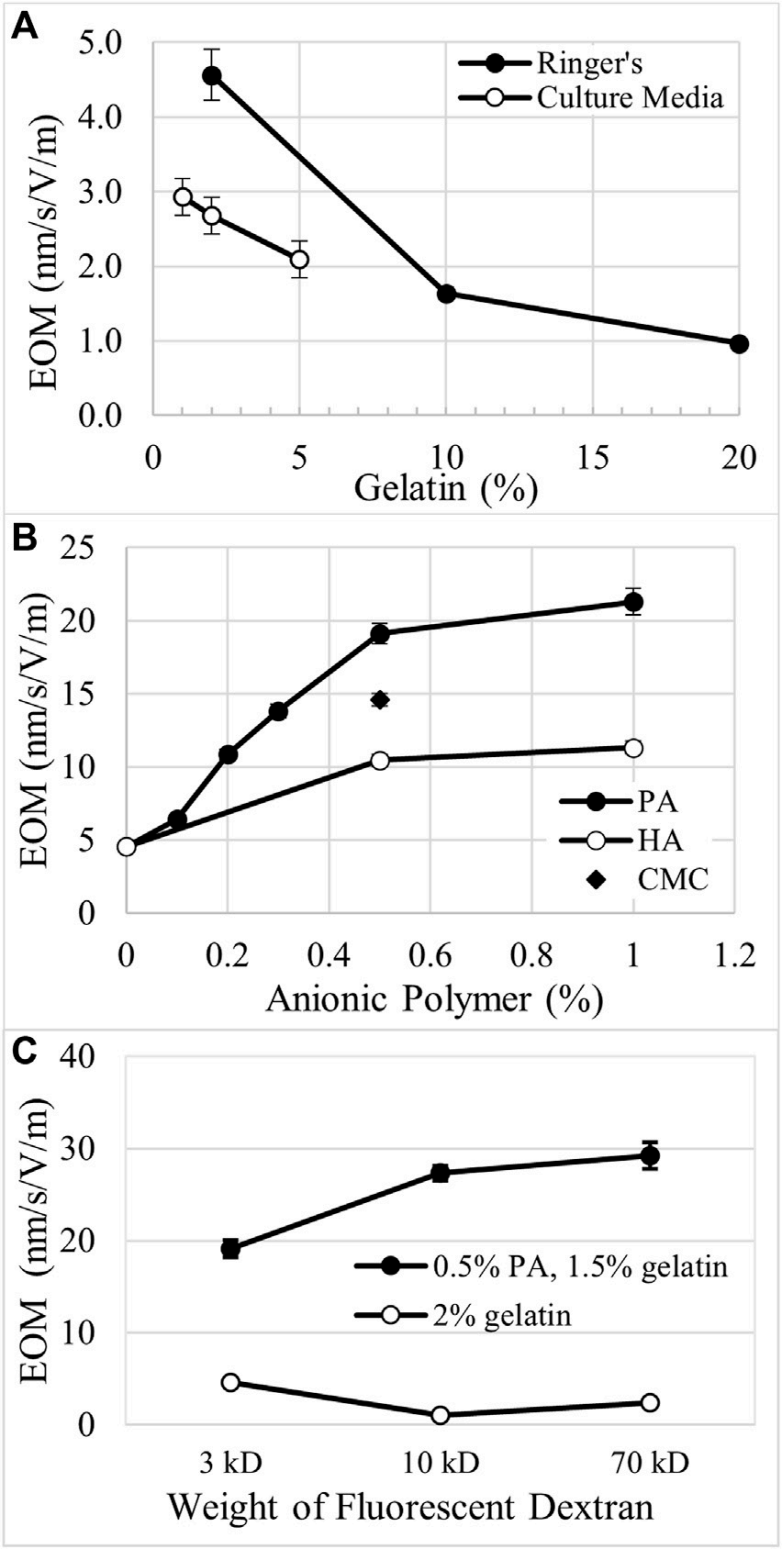
Comparison of electro-osmotic mobility (EOM) through different matrices. **(A)**. In gelatin matrices, EOM decreases with increasing concentration of gelatin when surrounded by either modified Ringer’s or culture media. **(B)**. Anionic polymers like polyacrylate (PA), hyaluronate (HA), and carboxymethyl cellulose (CMC) increase EOM in a concentration-dependent manner when mixed with gelatin. 2% was the total combined weight percent of the gelatin-polymer mixtures. **(C)**. EOM decreases for higher molecular weight dextrans in 2% gelatin, while EOM increases for the larger dextrans in 1.5%:0.5%, gelatin:PA.

As the electrical resistance of matrix-filled channels varied with matrix composition, current clamp generated electric fields of proportionally different strength. To account for the variation in the electric field strength, electro-osmotic mobility (EOM) was calculated for each matrix after the final EF strength was determined for each condition, i.e. EOM is EOF/applied EF. EOM of neutral Texas Red dextran (3 kD) was determined in gelatin matrices surrounded by modified Ringer’s or culture media. In 2% gelatin matrices, EOM was 4.6 nm/s/V/m and decreased by 64% and 79% in 10% and 20% gelatin, respectively, Figure 3A. In 2% gelatin surrounded by culture media, EOM was reduced by 41% compared to modified Ringer’s. Gelatin also reduced EOM in a concentration-dependent manner in culture media, Figure 3A.

Addition of anionic polymers to gelatin increases EOM in a concentration- and charge-dependent manner. The total amount of matrix was held constant at 2% by weight. High molecular weight HA possesses the lowest charge to mass ratio of the anionic polymers tested. When mixed in the matrix at 0.5% and 1.0%, HA increased EOM by 129% and 149%, respectively, compared to the EOM of 2% gelatin alone, Figure 3B, i.e. 0% weight of anionic polymer. Carboxymethyl cellulose possesses a greater charge to mass ratio than HA and increased EOM by 220% when used at 0.5% in 1.5% gelatin, Figure 3B. PA has the greatest charge to mass ratio of the polymers tested and increases EOM by 319% and 367% when combined at 0.5% and 1%, respectively, with gelatin. At low density, < 0.5% PA, there appears to be a steep, linear increase in EOM with PA concentration, Figure 3B. Above 0.5% PA, EOM appears to approach an asymptote.

We also explored how EOM was changed in higher concentrations of gelatin to attempt to replicate conditions in native cartilage. Mixtures of gelatin and PA at ratios of 15%:5% or 10%:10%, produced EOM of 1.9 ± 0.07 nm/s/V/m (n = 3) and 2.6 ± 0.5 nm/s/V/m (n = 3), respectively. While these values are greater than EOM through 20% gelatin alone, 1.0 ± 0.03 nm/s/V/m, they are much lower than the EOM through matrices with 10-fold less material, i.e. 1.5%:0.5% gelatin:PA, 19.1 ± 0.7 nm/s/V/m, and 1%:1%, 21.3 ± 0.9 nm/s/V/m, Figure 3B.

We explored the movement of larger molecules through these matrices by measuring EOM with 10 kD and 70 kD neutral Texas Red dextrans, Figure 3C. In 2% gelatin alone, 10 kD and 70 kD dextrans indicate EOM that is 77% and 47% lower than determined with the 3 kD dextran. However, in 1.5%:0.5%, gelatin:PA the larger dextrans indicate EOM that is 43% and 53% greater than the EOM determined with the 3 kD dextran, Figure 3C. Comparing the EOM calculated for the different sizes of the neutral fluorescent dextrans, the EOM through 1.5%:0.5%, gelatin:PA is amplified to 4.2–12.2 fold the EOM in 2% crosslinked gelatin alone.

Considering that the anionic polymers enhance EOM in extracellular matrices, we hypothesize that lower applied voltage could generate similar rates of electrokinetic perfusion through 1.5%:0.5%, gelatin:PA than 2% gelatin alone. Therefore, EOF through anionic matrices could reduce cell mortality at lower voltage. Mortality of cells did not increase in the presence of PA and physiologically relevant growth conditions. However, mixing cells into more concentrated matrices with higher viscosity did increase mortality measured at day 0, 14.0 ± 1.1% in 2% collagen, compared to lower concentration matrices, 5.4 ± 0.5% in ∼0.5% Matrigel (Sarkar and Messerli, 2021). Therefore, experimentation was limited to 2% matrices.

**FIGURE 4:**
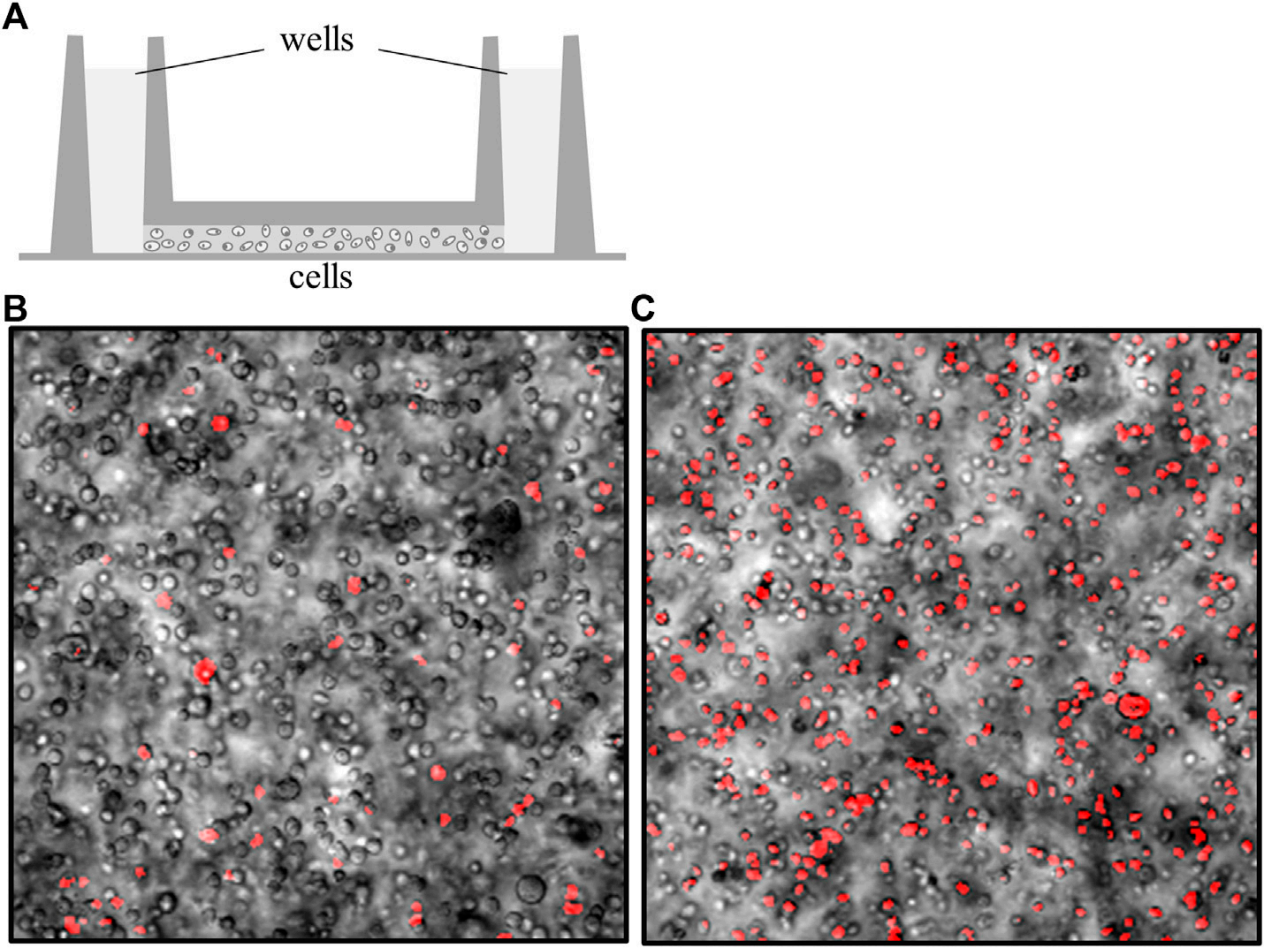
Cell mortality increases in the absence of interstitial flow. **(A)** Cells were mixed in artificial matrices and loaded into IBIDI channel slides (side view). A well on each side is filled with culture media that is continuous with fluid-filled adapters (not shown) (Sarkar and Messerli, 2021). The channel that spans between both wells is filled with a high density of cells embedded in different matrices. **(B)** Overlay of cells (transmitted light) with cells stained with propidium iodide (pseudo-colored red) to identify the necrotic cells. This image was acquired immediately after loading, gelling the matrix, and staining the cells in a 2% gelatin matrix, day 0. **(C)** Overlay of transmitted light cells with propidium iodide-stained cells after 48 h, in the absence of perfusion. Under these conditions, cell mortality increases by a factor just greater than 3, on average.

**FIGURE 5:**
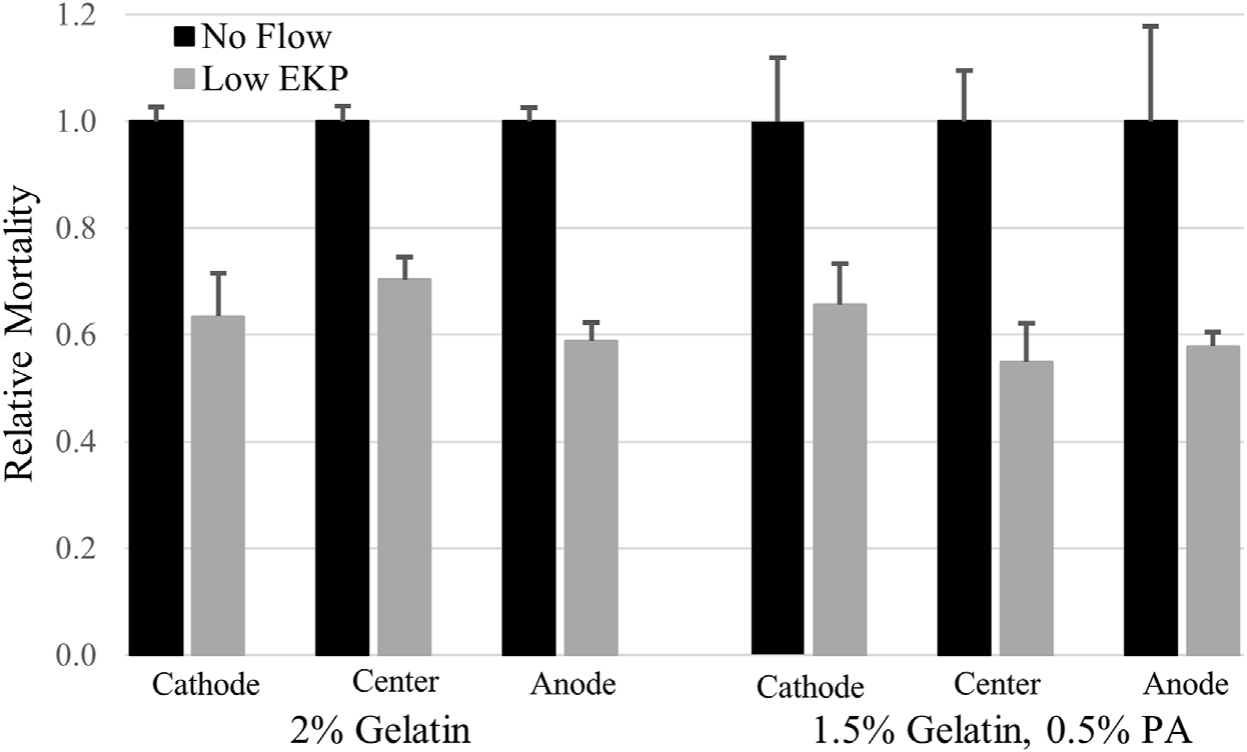
Low rates of electrokinetic perfusion (EKP) through 3D cultures reduced cell mortality. Current density of 32.4 mA/cm^2^ generated an EOF rate of 0.7 μm/s in the low charged 2% gelatin and significantly reduced mortality in all three regions of the 1.7 cm long channel (*p* < 0.001 for all three regions). Current density of only 5.8 mA/cm^2^ generated an EOF rate of 0.6 μm/s in the 1.5%:0.5%, gelatin:PA mixture and significantly reduced cell mortality (*p* < 0.03 cathode, *p* < 0.003 center, *p* < 0.04 anode) compared to the cells in the absence of flow.

Electrokinetic perfusion through 2% crosslinked gelatin was clamped, on average, to 32.4 mA/cm^2^ to attain an electric field of 251 mV/mm and an EOF of 0.7 μm/s. In the absence of EOF, cells showed a 3.1 fold increase in mortality, on average, after 48 h, Figure 4. In the presence of electrokinetic perfusion of the 2% gelatin, mortality was significantly reduced to 64%, on average, of the mortality in the absence of flow, Figure 5, (*p* < 0.001 for all three regions). To maintain a similar flow rate in the 1.5%:0.5%, gelatin:PA mixture, current was reduced to 5.8 mA/cm^2^, equivalent to an EF of 43.4 mV/mm and an average EOF of 0.6 μm/s. Even though the current density was decreased by a factor of 5.6, cell mortality in the gelatin:PA mixture significantly decreased to 59% of mortality, on average, under the same conditions in the absence of flow, Figure 5 (*p* < 0.03 cathode, *p* < 0.003 center, *p* < 0.04 anode). Reduction of cell mortality by the lower applied voltage through the anionic matrix was not statistically different from the reduced mortality achieved in gelatin alone at the higher applied potential at any of the three regions of the channels that were monitored (*p* > 0.5 cathode, *p* > 0.5 center, *p* > 0.5 anode, two-tailed *t*-test).

**FIGURE 6:**
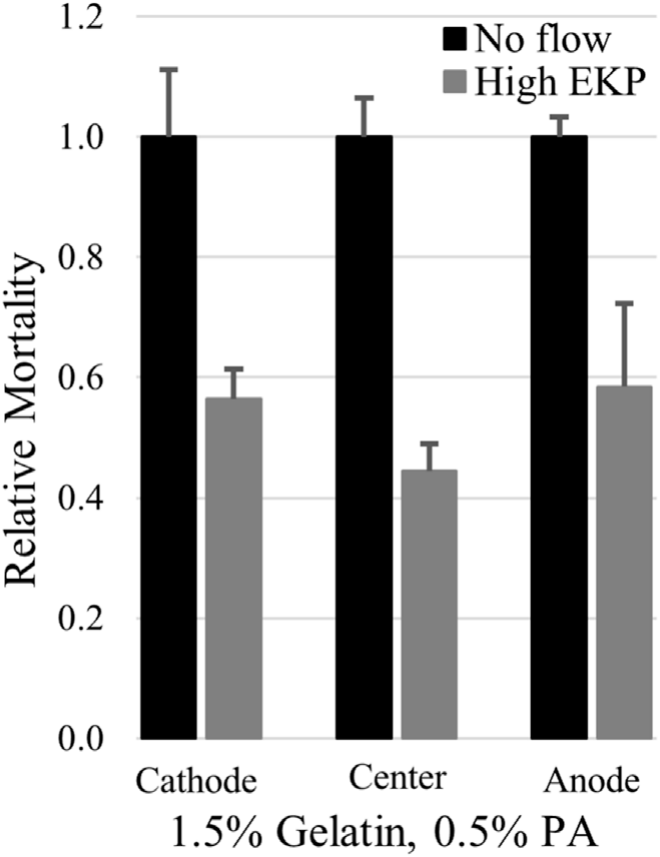
High rates of electrokinetic perfusion decreased cell mortality. At a current density of 100.7 mA/cm^2^ EOF was maintained at 9.8 μm/s and significantly reduced mortality (*p* < 0.005 cathode, *p* < 0.001 center, *p* < 0.02 anode) compared to the cells in the absence of flow.

Due to the amplified EOF, the upper limits of electrokinetic perfusion can be explored using lower, safer voltages with less concern for overheating of the sample. We were able to increase EOF to 9.8 μm/s in gelatin:PA and found that average cell mortality still decreased to 53%, on average, of mortality in the absence of flow, Figure 6 (*p* < 0.005 cathode, *p* < 0.001 center, *p* < 0.02 anode). However, the reduction of mortality at the higher flow rate was not significantly different from the reduction in mortality at the lower flow rate 0.6 μm/s (*p* > 0.17 cathode, *p* > 0.12 center, *p* > 0.48 anode, using a one-tailed *t*-test). Higher voltage used to induce EOF ≥15 μm/s, broke apart the gelatin:PA matrices before the end of the 48 h experiment.

### Thermal effects of applied EFs

Overheating of the experimental samples is a concern with electrokinetics. Modeling was first performed to estimate the temperature changes that may have occurred in the narrow IBIDI channels that were mounted on aluminum blocks held at 37°C. While the higher current densities were sufficient to increase heat within the matrix-filled channel, the heat rapidly dissipated from the low volume channel to the adjacent aluminum block so that the modeled temperature in the center of the matrix at the greatest field strength used in Figure 6 increased by less than 0.1 K. This result encouraged us to explore the possibility of overheating during electrokinetic perfusion *in vivo* or *in vitro* during tissue engineering, when a poorer thermal conductor surrounds the region with current flow.

**FIGURE 7:**
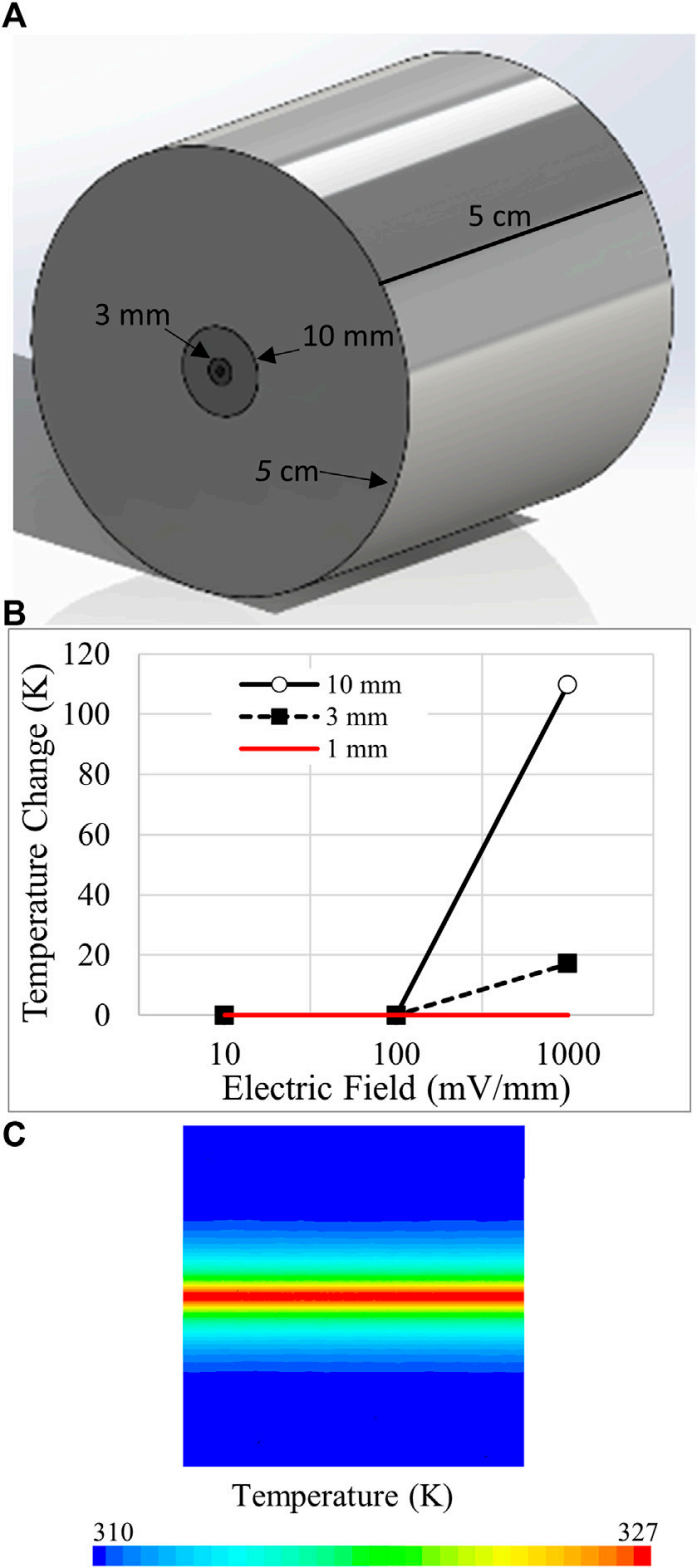
Modeling of temperature changes due to electrokinetic perfusion through narrow channels. **(A)** Ohmic heating during electrokinetic perfusion was constrained to small cylindrical regions of 1, 3, and 10 mm diameter and allowed to dissipate into a larger 5 cm diameter cylinder with thermal conductivity of 0.21 W/(m•K) to match cartilage. The length of the cylinder is also 5 cm. **(B)** Summary of the peak steady state heat for three regions of different diameter at 10, 100, and 1,000 mV/mm. DC current flow begins to generate damaging heat above 100 mV/mm. **(C)** Pseudo-colored heat map of the 3 mm cylinder at 1,000 mV/mm. The core of the cylinder reaches just above 327 K at steady state.

In the absence of local blood flow, electrokinetic perfusion could generate sufficient heat to be damaging or even lethal to cells, especially in stationary tissues. To explore these possibilities, we modeled heat dissipation from narrow constrained regions of electrokinetic perfusion into soft, avascular tissues, Figure 7A. Current flow through interior cylinders of increasing diameter, 1, 3 and 10 mm, was allowed to dissipate into the larger outer cylinder. The periphery of the large outer cylinder was modeled to possess the closest blood flow and was therefore, clamped to 37°C. In the smallest 1 mm diameter cylinder, electric fields ≤1,000 mV/mm DC did not accumulate heat greater than 0.1 K, Figure 7B. Using the same current densities through a larger region of tissue, 3 mm, the 1,000 mV/mm generated peak, steady state heat that reached 17 K above body temperature, Figure 7B. The heat generated under this condition initially increased at an average rate of 0.3 K/s for the first second of current flow. The heat map for a cross-section of the 3 mm diameter region is shown in Figure 7C for the 1,000 mV/mm field strength. In the largest diameter region, 10 mm, the 1,000 mV/mm field generated over 109 K but did not generate heat greater than 0.3 K above the starting temperature at 100 mV/mm. Under these conditions, heat also increased at an initial average rate of 0.3 K/s for the first second, in the central region of current flow. Dissipation of heat is significantly slower and dependent on volume. After 1 s of current flow at 1,000 mV/mm, heat dissipation for the 1, 3, and 10 mm regions required 3.1, 14.0 and 113.7 s to reduce the temperature 50% back to the starting temperature. Therefore, continuous DC electrokinetic perfusion could increase mortality due to hyperthermia, 40–46°C (Roti Roti, 2008) when applied using higher voltages and to regions of greater cross-sectional area.

## Discussion

Interstitial flow promotes mass transfer and also stimulates fibroblasts and chondrocytes to proliferate and differentiate as well as elaborate extracellular matrix molecules including collagen, glycosaminoglycans, and proteoglycans (Dan et al., 2010; Sharifi and Gharravi, 2019). Stimulating interstitial flow could therefore be a primary therapy to promote repair of soft tissues and their extracellular matrices. Electrokinetic perfusion may be a sufficient alternative to overcome the limitations of pressure-driven perfusion, but extracellular matrices must possess a sufficient amount of immobilized charge. While collagen is a primary extracellular matrix protein providing mechanical support to tissues and organs, we show that increasing concentrations of the relatively uncharged collagen increases electrical resistance and requires higher applied voltages to maintain EOF to reduce mortality. Increasing concentrations of charged polymers increases EOF and reduces mortality. In fact, we show that native charged polymers amplify EOF, enabling lower voltages to reduce mortality to the same extent as higher voltages applied to collagen gels. In the presence of charged matrices the applied voltages can be reduced even further below the sensory threshold of excitable cells.

### Electrical charge on collagen

The strength of collagen can be enhanced through crosslinking *in vitro* and *in vivo*, but it possesses weak charge density near physiological pH, Table 1, even after removal of amines from lysine during post-translational crosslinking with lysyl oxidase (Tanaka et al., 1981; Yamauchi and Sricholpech, 2012; Mesquida et al., 2018). Manufacturing of type A gelatin from collagen, used in this work, produces a product with an isoelectric point near pH 7.0–7.5, that more closely mimics the weak charge of native collagen than Type B gelatin with an isoelectric point near pH 5 (Ahsan and Rao, 2017). Covalent crosslinking of gelatin is required to enhance matrix strength as electrokinetic perfusion causes matrices to fracture in its absence. Cross-linking of gelatin with microbial transglutaminase is similar to cross-linking of collagen by lysyl oxidase *in vivo*, except for the site specific crosslinking by lysyl oxidase (Eyre et al., 2008). A side effect of microbial transglutaminase is that it removes positively charged amines and reduces the isoelectric point of gelatin (Dong et al., 2021). Therefore, the surface charge of the cross-linked gelatin used in these experiments, is predicted to be more negative than the cross-linked collagen *in vivo*. As a result, native collagen in physiological conditions and in the absence of proteoglycans or glycosaminoglycans, is likely to possess lower EOF than the crosslinked gelatin used in these experiments.

### Electrical resistance of matrices

Electrical resistance of the matrices varied with composition. Increasing the density of collagen alone, increased electrical resistance, Figure 2A, as the cross-sectional area for current flow decreased. However, by increasing the anionic polymers, the electrical resistance of the matrices decreased, Figures 2B,C, as the mobile counter ions were attracted to the polymers and increased their local concentration. This is consistent with the 2–10 fold increase in cations, i.e. Na^+^, K^+^ and Ca^2+^, and osmolarity found in articular cartilage (Hall et al., 1996). We conclude that the anionic polymers promote current flow through ECM compared to ECM comprised of relatively uncharged proteins alone.

### Modifiers of electrokinetic perfusion

The EOM rates measured in the presence of serum containing culture media are ∼40% lower than EOM measured in modified Ringer’s, Figure 3A. This is most likely due to the presence of macromolecules in the culture media. Chinese hamster ovary cells were used in these experiments due to their well characterized requirements in culture (Rasmussen et al., 1998; Ahn and Antoniewicz, 2011). However, their requirement for growth factors, included at lower cost by adding bovine serum, introduces other serum proteins that do not normally exist in the extracellular space of tissues. For example, lipids are known to block interstitial flow in extracellular matrices (McCarty et al., 2008). Therefore, it is likely that serum proteins that carry fats or cholesterol, e.g. lipoproteins or albumin, may be releasing fats that are adhering to regions of the ECM or the serum proteins themselves are adhering to the ECM. This would restrict flow or block charge on the ECM and reduce EOM (Lengyel and Guttman, 1999). As the extracellular space *in vivo* contains significantly lower amounts of soluble proteins, we hypothesize that the EOM values in native extracellular matrices will be relatively higher, similar to the flow rates measured in modified Ringer’s in the absence of soluble proteins, Figure 3A.

Addition of anionic polymers to the gelatin increases EOM that is dependent on the concentration of the polymer and the charge to mass ratio of the polymer, Figure 3B. PA increased EOM in a steep, linear manner up to 0.5% as the anionic PA replaced the relatively uncharged gelatin. Above 0.5%, PA was less effective in increasing EOM. We hypothesize that this is, in part, due to the increasing overlap of the electrical double layers (Wan, 1997). According to this hypothesis, the 0.5% PA is less efficient in generating EOM than native proteoglycans and glycosaminoglycans. In this work, the EOF/current density ratio determined for the 3 kD dextran through 1.5%:0.5%, gelatin:PA is 12.3 • 10^–9^ m^3^/A•s and is similar to the value for electrokinetic perfusion of tritiated water through thawed bovine articular cartilage, 7.5–12.8 • 10^–9^ m^3^/A•s (Garcia et al., 1996) that contains about ten times greater density of matrix macromolecules. Our attempts at generating EOF through similar densities, 10%:10% gelatin:PA, generated values that were nearly 10 times lower, i.e. 1.6 • 10^–9^ m^3^/A•s. The higher density of low charged gelatin increased resistance to flow that was not offset by the increase in anionic polymers. This may be due to two different factors. First, the proteoglycans and glycosaminoglycans possess lower charge to mass ratios than PA. *In vivo,* the glycans are well organized and spaced apart on the proteoglycans. For example, the structure of the proteoglycan, aggrecan, is referred to as the ‘bottle brush’ due to its linear protein structure and regular spacing of anionic keratan sulfate and chondroitin sulfate chains surrounding the protein core like the bristles extending from the brush (Chandran and Horkay, 2012). The proteoglycans are also spaced apart on HA (Sophia Fox et al., 2009). The random distribution of the anionic polymers in the matrices of this work allowed greater overlap of the electrical double layers and reduced efficiency for generating EOM at lower concentrations. The three-dimensional structure of the bottle brush configuration of proteoglycans *in vivo* may also have increased porosity and reduced tortuosity compared to the linear anionic polymers used in this study. We conclude that construction and use of bottle brush polymers (Li et al., 2021) with anionic charge should increase efficiency and have substantial impact on tissue engineering applications.

Large neutral fluorescent tracers displayed different EOM trends in the matrices. In 2% gelatin, the 10 kD and 70 kD fluorescent dextrans reflected significantly lower EOM than the 3 kD dextran. However, in the presence of 1.5% gelatin with 0.5% PA, the 10 kD and 70 kD fluorescent dextrans reflected significantly higher EOM than the 3 kD dextran, Figure 3C. This could be due to a difference in tortuosity or pore size between the charged and uncharged matrices. Tortuosity for a 3 kD dextran was reported to be near unity for both uncharged and charged gels (Faraji et al., 2011). However, tortuosity was higher for a 70 kD dextran in neutral acrylamide gels compared to gels made with greater negative charge. Also, crosslinking gelatin with microbial transglutaminase is reported to lead to smaller pore size compared to gelatin in the absence of enzyme, and also crosslinking of gelatin in the presence of cellulose nanocrystals (Dong et al., 2021). The anionic polymers may be interfering with the amount of gelatin crosslinking performed by the enzyme leading to larger pores. Glycosaminoglycans and proteoglycans may be influencing packing and crosslinking of ECM proteins *in vivo*.

The more highly charged matrix amplifies EOM compared to gelatin alone, Figure 3B. Therefore, a current density that was 5.6-fold lower in the anionic matrix increased flow and significantly reduced mortality to the same extent as the higher current density in the 2% gelatin matrix. By reducing the applied voltage in the charged matrix, electrophoresis of macromolecules was also reduced while electro-osmosis was maintained. This shift in the relative strength of the two forces supports the importance of electro-osmosis over electrophoresis in promoting cell viability in the presence of an electric field. In an earlier report, we reduced cell mortality in ∼0.5% Matrigel extracellular matrix (Sarkar and Messerli, 2021). Matrigel is comprised of a couple thousand unique proteins including a few proteoglycans (Hughes et al., 2010) and possesses significantly lower EOM at ∼0.5%, i.e. 7.4 nm/s/V/m (Sarkar and Messerli, 2021), than the 1.5% gelatin, 0.5% PA mixture used here, i.e. 19.1 nm/s/V/m in Ringer’s, indicating that Matrigel possesses a relatively lower average net charge.

The relationship between anionic matrices and EOM has interesting implications for endogenous electric fields. In a first example, the injury current near epidermal wounds generates electric fields around 100 mV/mm (Barker et al., 1982). These fields were considered to promote electro-osmosis based on the surface potential of the keratinocytes (Nishimura et al., 1996). However, the keratinocytes in the basal and spinous layers of the deeper epidermis are surrounded by a high density of HA (Tammi et al., 1988; Anderegg et al., 2014). We hypothesize that the high concentration of HA between these cells amplifies local EOF in the epidermis during injury, well above that generated by the surface charge of keratinocytes alone. In a second example, the studies reported here used isotropic gels in uniform channels. However, *in vivo*, changes in local geometry or charge density could give rise to more complex flow fields and enhance mixing (Gaikwad et al., 2020; Mehta et al., 2021).

### Comparison of electrokinetic perfusion with pressure-driven perfusion

Pressure-driven perfusion has been challenging for generating interstitial flow due to the increase in resistance to flow in narrow spaces. Enhancing porosity of 6.4% gelatin gels by making fibrous strands into a macroporous hydrogel enhanced survival during pressure-driven perfusion but appeared to weaken the matrix (Chen et al., 2014). Pressure-driven perfusion through 0.2% matrix is possible when supported by porous supporting structures at the inlet and outlet of the perfusion chamber (Ng and Swartz, 2003), or through 0.5% Matrigel supported by agarose at the inlet and crosslinked to the enclosing walls of the perfusion chamber (Sarkar and Messerli, 2021). Higher density ECMs like those found *in vivo,* ∼10–20% dry weight, will make pressure-driven perfusion through tissues even more challenging.

Pressure-driven perfusion through artificial matrices at rates >13 μm/s is reported to damage fibroblasts (Ng and Swartz, 2006). We attempted to test the effects of this higher rate of flow using electrokinetic perfusion but only reached 9.8 μm/s before matrices were not able to maintain their integrity in the open IBIDI channels. Cell mortality measured when cells were exposed to 9.8 μm/s flow rate was not significantly different compared to cells exposed to 0.6 μm/s. However, there is significantly lower mortality at 1.5 μm/s than 0.4 μm/s flow rate (Sarkar and Messerli, 2021). This may indicate an upper limit for flow rate in reducing cell mortality. Interstitial flow is predicted to reach 2 μm/s per second *in vivo* (Swartz and Fleury, 2007). Flow rates higher than this may not have an effect in reducing mortality especially in small tissues. However, in larger engineered tissues without vasculature, higher interstitial flow rates may be useful to ensure nutrients are reaching deeper cells. To reach the higher rates of flow we would need to promote greater cross-linking of the protein matrix and could add more anionic polymers or more well-organized anionic polymers to reduce overlap of the electrical double layers.

### Future guidance

These efforts have helped to identify two potentially limiting factors for implementing electrokinetic perfusion. First, ohmic heating appears that it will damage cells when the EFs rise above 100 mV/mm. Our model made use of DC EFs that generated rises in heat of 0.3 K/s at 1,000 mV/mm. These temperature changes could be easily overcome by pulsing the EFs for fractions of seconds with delays between the pulses as performed in the clinics (Kloth, 2014). EOF reaches steady state very rapidly (Soderman and Jonsson, 1996; Sarkar and Messerli, 2021) compared to the thermal changes modeled here. We hypothesize that pulsed EFs will generate directional EOF in native tissues and that backflow will be prevented by resistance to pressure-driven flow. Additional modeling would be useful to initially test this hypothesis and determine the true electric field strength in tissues when current is not constrained. The second limit appears to be viscosity of the extracellular matrices. The larger molecular weight anionic polymers were used in these experiments to prevent them from migrating out of the matrix during the 48 h experiment. However, the longer polymers increase viscosity that was damaging to cells. Smaller anionic polymers would be more useful if they could be cross-linked into the matrix. Careful construction of more concentrated matrices with cells may be achieved by layering of cells with matrix materials using modern tissue printing devices (Noor et al., 2019).

We conclude that the native charged extracellular matrix molecules are enhancing electrokinetic perfusion during electrical stimulation. Considering that interstitial flow promotes supporting functions of soft tissues (Dan et al., 2010; Sharifi and Gharravi, 2019), electrical stimulation might be altered to optimize electrokinetic perfusion so that it more closely mimics the natural, steady or intermittent interstitial flows that occur in different connective tissues or epithelia. Electrokinetic perfusion may also be useful for promoting interstitial flow and increasing cell viability during tissue engineering or transplantation prior to vascularization.

## Data Availability

The original contributions presented in the study are included in the article/supplementary material, further inquiries can be directed to the corresponding author.
